# (*E*)-1-(3,4-Dimethyl­benzyl­idene)-2,2-diphenyl­hydrazine

**DOI:** 10.1107/S1600536811015352

**Published:** 2011-05-07

**Authors:** Angel Mendoza, Blanca M. Cabrera-Vivas, Ruth Meléndrez-Luevano, Juan C. Ramírez, Marcos Flores-Alamo

**Affiliations:** a Centro de Química, ICUAP, Benemérita Universidad Autónoma de Puebla, Puebla, Pue., Mexico; b Facultad de Ciencias Químicas, Benemérita Universidad Autónoma de Puebla, Puebla, Pue., Mexico; c Facultad de Química, Universidad Nacional Autónoma de México, 04510 México DF, Mexico

## Abstract

The asymmetric unit of the title compound, C_21_H_20_N_2_, contain two mol­ecules, both of them showing an *E* configuration of the C=N bond. The dihedral angles between the phenyl rings in the phenyl­hydrazone groups are 86.84 (10) and 84.85 (8)° for the two mol­ecules. Inter­molecular C—H⋯π inter­actions are observed in the crystal structure.

## Related literature

For applications of hydrazones, see: Angell *et al.* (2006 ▶); Buss *et al.* (2004 ▶); Melnyk *et al.* (2006 ▶); Ranford *et al.* (1998 ▶). For related structures see: Clulow *et al.* (2008 ▶); Mendoza *et al.* (2010 ▶).
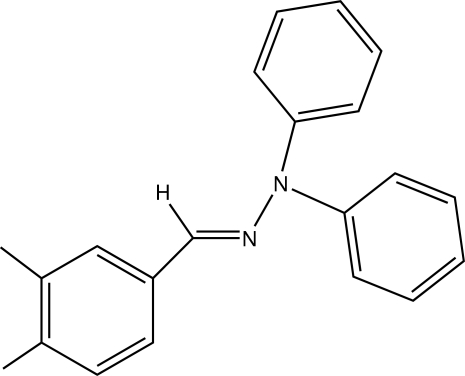


## Experimental

### 

#### Crystal data

C_21_H_20_N_2_
*M* *_r_* = 300.39
Triclinic, 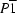

*a* = 9.9375 (5) Å
*b* = 10.6322 (5) Å
*c* = 17.5680 (8) Å
α = 77.530 (4)°
β = 76.480 (4)°
γ = 77.074 (4)°
*V* = 1732.60 (14) Å^3^
*Z* = 4
Mo *K*α radiation
μ = 0.07 mm^−1^
*T* = 293 K
0.61 × 0.42 × 0.27 mm

#### Data collection

Oxford Diffraction Xcalibur Atlas Gemini diffractometer
Absorption correction: analytical (*CrysAlis PRO*; Oxford Diffraction, 2009 ▶) *T*_min_ = 0.971, *T*_max_ = 0.984
12246 measured reflections
6288 independent reflections
3202 reflections with *I* > 2σ(*I*)
*R*_int_ = 0.027

#### Refinement

*R*[*F* ^2^ > 2σ(*F* ^2^)] = 0.040
*wR*(*F* ^2^) = 0.100
*S* = 0.85
6288 reflections
420 parameters
H-atom parameters constrained
Δρ_max_ = 0.15 e Å^−3^
Δρ_min_ = −0.11 e Å^−3^

### 

Data collection: *CrysAlis CCD* (Oxford Diffraction, 2009 ▶); cell refinement: *CrysAlis RED* (Oxford Diffraction, 2009 ▶); data reduction: *CrysAlis RED*; program(s) used to solve structure: *SHELXS97* (Sheldrick, 2008 ▶); program(s) used to refine structure: *SHELXL97* (Sheldrick, 2008 ▶); molecular graphics: *ORTEP-3 for Windows* (Farrugia, 1997 ▶); software used to prepare material for publication: *WinGX* (Farrugia, 1999 ▶).

## Supplementary Material

Crystal structure: contains datablocks global, I. DOI: 10.1107/S1600536811015352/bt5512sup1.cif

Structure factors: contains datablocks I. DOI: 10.1107/S1600536811015352/bt5512Isup2.hkl

Additional supplementary materials:  crystallographic information; 3D view; checkCIF report

## Figures and Tables

**Table 1:** Hydrogen-bond geometry (Å, °) *Cg*1, *Cg*2, *Cg*3 and *Cg*4 are the centroids of the C31–C36, C37–C42, C2–C7 and C23–C28 rings, respectively.

*D*—H⋯*A*	*D*—H	H⋯*A*	*D*⋯*A*	*D*—H⋯*A*
C3—H3⋯*Cg*1^i^	0.93	2.81	3.7152 (18)	166
C6—H6⋯*Cg*1^ii^	0.93	2.66	3.5506 (19)	160
C9—H9*C*⋯*Cg*2^iii^	0.96	2.97	3.7170 (19)	136
C29—H29*B*⋯*Cg*3^iv^	0.96	3.00	3.931 (2)	165
C41—H41⋯*Cg*4^i^	0.93	2.83	3.590 (2)	139

Symmetry codes: (i) 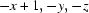; (ii) 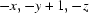; (iii) 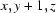; (iv) 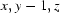.

**Fig. 1.**
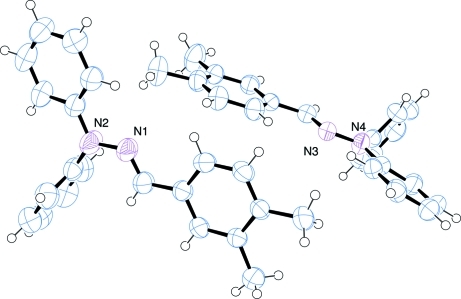
The molecular structure and the atom labelling scheme for I. Displacement ellipsoids are draw at the 50% probability level and H atoms are shown as circles of arbitrary size.

## supplementary crystallographic information

### Comment

Many applications are known for hydrazones and their derivatives in the chemical analysis field.  Employing   these compounds as molecular sensors in determination and quantization of aldehydes and ketones in gas currents allows their use in environmental, biological and industrial applications (Angell, *et al.* 2006). Hydrazones have been used in the treatment of several diseases as malaria (Melnyk *et al.*, 2006) or genetic disorders (Ranford *et al.*, 1998). Coordination compounds with iron have shown therapeutic attributes in the treatment of cancer (Buss *et al.*, 2004).

The title compound I, C_21_H_20_N_2_, presents an E configuration of the C=N double bond. The asymmetric unit contains two non-planar molecules. The dihedral angles between the C10/C11/C12/C13/C14/C15 ring and C16/C17/C18/C19/C20/C21 ring is 86.84 (10)° for molecule 1 (N1 to C21). The dihedral angle for the phenyl rings C31/C32/C33/C34/C35/C36 and C37/C38/C39/C40/C41/C42 is 84.85 (8)° for molecule 2 (N3 to C42). The dimethyl-phenyl rings are slightly twisted with respect to the C=N group with torsion angles of 2.8 (2)° for N1/C1/C2/C7 and of 1.1 (2)° for N3/C22/C23/C24. The N—N distances [N1—N2 1.3765 (17) Å and N3—N4 1.3701 (16) Å] are shorter than found in free diphenylhydrazine [1.418 (2) Å] (Clulow, *et al.*, 2008). The imine bond distances [N1—C1 1.277 (2) Å and N3—C22 1.2797 (18) Å] are longer than N=C typical bond and shorter [1.287 (2) Å] than related structures with *N*,*N*-diphenylhidrazone group (Mendoza *et al.* 2010). Intermolecular C—H···π interactions are observed.

### Experimental

*N*,*N*-diphenylhydrazine (592 mg, 2.68 mmol) was dissolved in ethanol and acetic acid (0.5 ml) was added slowly into this solution while stirring. 300 mg (2.24 mmol) of 3,4-dimethylbenzaldehyde was added drop by drop into the above solution with strong stirring and the resulting mixture was kept at atmospheric temperature until it became amber transparent solution. After three hours the amber solution turns to be precipitated. The mixture was separated with filtration in vacuum system and the precipitate was washed three times with cold methanol. Recrystallization was performed several times with acetonitrile, to obtain colorless crystals for X-ray analysis. Yield 72% at 25°C, m. p. 95–99°C. UV λ_max_ = 341.24 nm. FT. IR (film): (cm^-1^): 3033 ν(C—H), 2933 ν(C—H), 1588, 1490 ν(C=N), 1221 ν(C=N—N). ^1^H NMR (400 MHz (CD_3_)_2_CO: (δ p.p.m.): 7.46–7.41 (m, 4H), 7.37 (s, 1H), 7.35–7.32 (dd, 1H), 7.22–7.16 (m, 6H), 7.13 (s, 1H), 7.10–7.08 (d, 1H), 2.23, 2.21 (2 s, 6H). ^13^C NMR (400 MHz, (CD_3_)_2_CO: (δ p.p.m.): 143.83, 136.72, 136.56, 135.72, 133.90, 129.84, 129.77, 127.45, 124.41, 123.78, 122.35, 18.91, 18.81. MS—EI: m/*z* = 300 *M*^+^.

### Refinement

H atoms  were placed in geometrically idealized positions, and refined as riding on their parent atoms, with C—H distances fixed to 0.930 Å (aromatic CH) with *U*iso = 1.2Ueq(C), 0.960 Å (methyl CH_3_) with *U*iso = 1.5Ueq(C).

### Crystal data

**Table:**

C_21_H_20_N_2_	*Z* = 4
*M**_r_* = 300.39	*F*(000) = 640
Triclinic, *P*1	*D*_x_ = 1.152 Mg m^−^^3^
*a* = 9.9375 (5) Å	Mo *K*α radiation, λ = 0.71073 Å
*b* = 10.6322 (5) Å	Cell parameters from 3720 reflections
*c* = 17.5680 (8) Å	θ = 3.4–25.2°
α = 77.530 (4)°	µ = 0.07 mm^−^^1^
β = 76.480 (4)°	*T* = 293 K
γ = 77.074 (4)°	Prism, colorless
*V* = 1732.60 (14) Å^3^	0.61 × 0.42 × 0.27 mm

### Data collection

**Table:**

Oxford Diffraction Xcalibur Atlas Gemini diffractometer	6288 independent reflections
graphite	3202 reflections with *I* > 2σ(*I*)
Detector resolution: 10.4685 pixels mm^-1^	*R*_int_ = 0.027
ω scans	θ_max_ = 25.3°, θ_min_ = 3.4°
Absorption correction: analytical (*CrysAlis PRO*; Oxford Diffraction, 2009)	*h* = −9→11
*T*_min_ = 0.971, *T*_max_ = 0.984	*k* = −10→12
12246 measured reflections	*l* = −21→20

### Refinement

**Table:**

Refinement on *F*^2^	Secondary atom site location: difference Fourier map
Least-squares matrix: full	Hydrogen site location: inferred from neighbouring sites
*R*[*F*^2^ > 2σ(*F*^2^)] = 0.040	H-atom parameters constrained
*wR*(*F*^2^) = 0.100	*w* = 1/[σ^2^(*F*_o_^2^) + (0.0496*P*)^2^] where *P* = (*F*_o_^2^ + 2*F*_c_^2^)/3
*S* = 0.85	(Δ/σ)_max_ = 0.001
6288 reflections	Δρ_max_ = 0.15 e Å^−^^3^
420 parameters	Δρ_min_ = −0.11 e Å^−^^3^
0 restraints	Extinction correction: *SHELXL97* (Sheldrick, 2008), Fc^*^=kFc[1+0.001xFc^2^λ^3^/sin(2θ)]^-1/4^
Primary atom site location: structure-invariant direct methods	Extinction coefficient: 0.0220 (12)

### Special details

**Table:**

Geometry. All s.u.'s (except the s.u. in the dihedral angle between two l.s. planes) are estimated using the full covariance matrix. The cell s.u.'s are taken into account individually in the estimation of s.u.'s in distances, angles and torsion angles; correlations between s.u.'s in cell parameters are only used when they are defined by crystal symmetry. An approximate (isotropic) treatment of cell s.u.'s is used for estimating s.u.'s involving l.s. planes.
Refinement. Refinement of *F*^2^ against ALL reflections. The weighted *R*-factor *wR* and goodness of fit *S* are based on *F*^2^, conventional *R*-factors *R* are based on *F*, with *F* set to zero for negative *F*^2^. The threshold expression of *F*^2^ > 2σ(*F*^2^) is used only for calculating *R*-factors(gt) *etc*. and is not relevant to the choice of reflections for refinement. *R*-factors based on *F*^2^ are statistically about twice as large as those based on *F*, and *R*- factors based on ALL data will be even larger.

### Fractional atomic coordinates and isotropic or equivalent isotropic displacement parameters (Å^2^)

**Table:**

	*x*	*y*	*z*	*U*_iso_*/*U*_eq_	
N1	0.36159 (15)	0.39399 (14)	0.36518 (8)	0.0660 (4)	
N2	0.41791 (15)	0.32889 (15)	0.43054 (8)	0.0756 (4)	
C16	0.32092 (19)	0.29285 (16)	0.49984 (10)	0.0597 (4)	
C21	0.1779 (2)	0.33547 (18)	0.50422 (11)	0.0730 (5)	
H21	0.1443	0.3888	0.4607	0.088*	
C20	0.0850 (2)	0.2992 (2)	0.57287 (12)	0.0858 (6)	
H20	−0.0111	0.3292	0.5752	0.103*	
C19	0.1307 (3)	0.2199 (2)	0.63774 (12)	0.0875 (6)	
H19	0.0669	0.1956	0.6837	0.105*	
C18	0.2719 (3)	0.17699 (19)	0.63365 (11)	0.0812 (6)	
H18	0.3043	0.1226	0.6772	0.097*	
C17	0.3673 (2)	0.21310 (18)	0.56583 (10)	0.0730 (5)	
H17	0.4632	0.1838	0.5643	0.088*	
C10	0.56693 (18)	0.29103 (18)	0.42665 (9)	0.0601 (5)	
C11	0.6403 (2)	0.37197 (19)	0.44503 (11)	0.0787 (6)	
H11	0.5936	0.4524	0.4589	0.094*	
C12	0.7826 (3)	0.3344 (3)	0.44297 (12)	0.0907 (6)	
H12	0.8317	0.3887	0.4563	0.109*	
C13	0.8510 (2)	0.2186 (3)	0.42167 (12)	0.0936 (7)	
H13	0.9472	0.1932	0.4204	0.112*	
C14	0.7801 (3)	0.1397 (2)	0.40227 (13)	0.0963 (7)	
H14	0.8281	0.0604	0.3873	0.116*	
C15	0.6372 (2)	0.1753 (2)	0.40443 (11)	0.0806 (6)	
H15	0.5891	0.1204	0.3908	0.097*	
C1	0.44210 (18)	0.43109 (16)	0.29977 (10)	0.0617 (5)	
H1	0.5391	0.4145	0.2964	0.074*	
C2	0.38244 (17)	0.49919 (15)	0.23059 (9)	0.0532 (4)	
C7	0.23891 (17)	0.52161 (16)	0.23122 (10)	0.0606 (5)	
H7	0.1774	0.4926	0.2771	0.073*	
C6	0.18753 (17)	0.58661 (16)	0.16420 (10)	0.0617 (5)	
H6	0.0913	0.5999	0.1656	0.074*	
C5	0.27467 (16)	0.63270 (15)	0.09501 (9)	0.0513 (4)	
C4	0.41897 (16)	0.61329 (15)	0.09341 (9)	0.0518 (4)	
C3	0.46939 (16)	0.54615 (16)	0.16103 (10)	0.0572 (4)	
H3	0.5657	0.532	0.1597	0.069*	
C8	0.51750 (17)	0.66410 (19)	0.01989 (10)	0.0770 (5)	
H8A	0.613	0.6321	0.0267	0.115*	
H8B	0.4992	0.7581	0.0111	0.115*	
H8C	0.5032	0.6345	−0.025	0.115*	
C9	0.21465 (17)	0.70064 (17)	0.02243 (10)	0.0686 (5)	
H9A	0.1143	0.7069	0.0346	0.103*	
H9B	0.2544	0.6513	−0.0199	0.103*	
H9C	0.2371	0.7869	0.0063	0.103*	
N3	0.19466 (12)	0.19292 (13)	0.00863 (7)	0.0525 (3)	
N4	0.19869 (13)	0.19943 (13)	−0.07059 (7)	0.0565 (4)	
C31	0.18030 (15)	0.32507 (16)	−0.11695 (9)	0.0477 (4)	
C32	0.13244 (15)	0.43680 (16)	−0.08245 (9)	0.0522 (4)	
H32	0.1149	0.4297	−0.0275	0.063*	
C33	0.11102 (16)	0.55796 (17)	−0.12972 (10)	0.0599 (4)	
H33	0.0781	0.6322	−0.106	0.072*	
C34	0.13712 (17)	0.57179 (18)	−0.21115 (11)	0.0657 (5)	
H34	0.1215	0.6542	−0.2424	0.079*	
C35	0.18661 (18)	0.46180 (19)	−0.24540 (10)	0.0663 (5)	
H35	0.2056	0.4698	−0.3005	0.08*	
C36	0.20849 (17)	0.33961 (17)	−0.19914 (9)	0.0600 (4)	
H36	0.2426	0.266	−0.2233	0.072*	
C37	0.23353 (16)	0.08491 (15)	−0.10724 (8)	0.0492 (4)	
C38	0.12895 (17)	0.03792 (17)	−0.12529 (10)	0.0587 (4)	
H38	0.0355	0.078	−0.1116	0.07*	
C39	0.1620 (2)	−0.06805 (18)	−0.16345 (10)	0.0681 (5)	
H39	0.091	−0.0988	−0.1763	0.082*	
C40	0.2989 (2)	−0.12838 (17)	−0.18261 (10)	0.0704 (5)	
H40	0.3213	−0.2002	−0.2084	0.084*	
C41	0.4032 (2)	−0.08287 (19)	−0.16375 (10)	0.0749 (5)	
H41	0.4963	−0.1245	−0.1763	0.09*	
C42	0.37124 (17)	0.02402 (18)	−0.12631 (10)	0.0648 (5)	
H42	0.4425	0.055	−0.1139	0.078*	
C22	0.21859 (15)	0.08199 (17)	0.05388 (9)	0.0532 (4)	
H22	0.2346	0.0053	0.0333	0.064*	
C23	0.22101 (14)	0.07456 (16)	0.13727 (9)	0.0479 (4)	
C28	0.24973 (16)	−0.04682 (16)	0.18511 (9)	0.0544 (4)	
H28	0.2625	−0.1219	0.1633	0.065*	
C27	0.26025 (16)	−0.06090 (16)	0.26412 (9)	0.0572 (4)	
C26	0.23930 (16)	0.05158 (17)	0.29740 (9)	0.0575 (4)	
C25	0.20856 (17)	0.17242 (17)	0.25014 (10)	0.0628 (5)	
H25	0.1929	0.2477	0.2722	0.075*	
C24	0.20038 (16)	0.18513 (16)	0.17172 (10)	0.0591 (5)	
H24	0.181	0.2681	0.1416	0.071*	
C29	0.2983 (2)	−0.19570 (18)	0.31134 (11)	0.0935 (7)	
H29A	0.393	−0.2085	0.3194	0.14*	
H29B	0.2911	−0.2606	0.2827	0.14*	
H29C	0.235	−0.2038	0.3619	0.14*	
C30	0.2536 (2)	0.0441 (2)	0.38198 (10)	0.0887 (6)	
H30A	0.2308	0.1307	0.3948	0.133*	
H30B	0.3486	0.0058	0.3876	0.133*	
H30C	0.1904	−0.0089	0.4173	0.133*	

### Atomic displacement parameters (Å^2^)

**Table:**

	*U*^11^	*U*^22^	*U*^33^	*U*^12^	*U*^13^	*U*^23^
N1	0.0642 (9)	0.0726 (10)	0.0561 (9)	−0.0088 (7)	−0.0201 (8)	0.0053 (8)
N2	0.0601 (10)	0.0977 (12)	0.0557 (9)	−0.0060 (8)	−0.0172 (8)	0.0129 (8)
C16	0.0683 (12)	0.0599 (12)	0.0512 (11)	−0.0110 (9)	−0.0159 (10)	−0.0064 (9)
C21	0.0701 (13)	0.0872 (15)	0.0601 (12)	−0.0164 (10)	−0.0175 (10)	−0.0020 (10)
C20	0.0763 (13)	0.1129 (18)	0.0689 (14)	−0.0269 (12)	−0.0096 (12)	−0.0118 (13)
C19	0.1008 (18)	0.1049 (18)	0.0549 (13)	−0.0310 (13)	−0.0027 (12)	−0.0107 (12)
C18	0.1102 (18)	0.0804 (15)	0.0494 (12)	−0.0108 (13)	−0.0185 (12)	−0.0072 (10)
C17	0.0839 (13)	0.0763 (14)	0.0545 (12)	−0.0046 (10)	−0.0190 (11)	−0.0067 (10)
C10	0.0620 (12)	0.0657 (13)	0.0489 (10)	−0.0040 (10)	−0.0170 (9)	−0.0029 (9)
C11	0.0801 (15)	0.0813 (15)	0.0751 (13)	−0.0075 (12)	−0.0145 (11)	−0.0223 (11)
C12	0.0837 (17)	0.115 (2)	0.0818 (15)	−0.0300 (14)	−0.0284 (12)	−0.0101 (14)
C13	0.0675 (14)	0.121 (2)	0.0756 (15)	−0.0010 (15)	−0.0203 (11)	0.0084 (14)
C14	0.0977 (19)	0.0835 (17)	0.0968 (17)	0.0175 (14)	−0.0238 (14)	−0.0222 (13)
C15	0.0903 (16)	0.0723 (15)	0.0818 (14)	−0.0036 (12)	−0.0299 (12)	−0.0158 (11)
C1	0.0564 (11)	0.0663 (12)	0.0590 (11)	−0.0090 (9)	−0.0171 (9)	0.0007 (9)
C2	0.0523 (10)	0.0510 (11)	0.0562 (10)	−0.0111 (8)	−0.0165 (9)	−0.0005 (8)
C7	0.0534 (11)	0.0666 (12)	0.0596 (11)	−0.0198 (8)	−0.0100 (9)	0.0021 (9)
C6	0.0472 (10)	0.0696 (12)	0.0693 (12)	−0.0165 (8)	−0.0196 (9)	0.0009 (10)
C5	0.0527 (10)	0.0492 (10)	0.0564 (10)	−0.0152 (8)	−0.0194 (9)	−0.0025 (8)
C4	0.0519 (10)	0.0519 (11)	0.0541 (10)	−0.0164 (8)	−0.0148 (8)	−0.0020 (8)
C3	0.0451 (9)	0.0667 (12)	0.0603 (11)	−0.0117 (8)	−0.0192 (9)	−0.0011 (9)
C8	0.0620 (12)	0.1005 (15)	0.0667 (12)	−0.0268 (10)	−0.0176 (10)	0.0070 (11)
C9	0.0655 (11)	0.0738 (13)	0.0696 (12)	−0.0174 (9)	−0.0294 (9)	0.0034 (9)
N3	0.0526 (8)	0.0588 (10)	0.0472 (8)	−0.0148 (6)	−0.0124 (6)	−0.0035 (7)
N4	0.0715 (9)	0.0548 (10)	0.0448 (8)	−0.0116 (7)	−0.0162 (7)	−0.0065 (7)
C31	0.0430 (9)	0.0545 (11)	0.0468 (10)	−0.0125 (7)	−0.0134 (7)	−0.0023 (8)
C32	0.0488 (10)	0.0586 (12)	0.0495 (10)	−0.0082 (8)	−0.0143 (8)	−0.0066 (9)
C33	0.0588 (11)	0.0573 (12)	0.0652 (12)	−0.0074 (8)	−0.0224 (9)	−0.0065 (9)
C34	0.0697 (12)	0.0599 (13)	0.0661 (13)	−0.0175 (9)	−0.0227 (10)	0.0079 (10)
C35	0.0762 (13)	0.0729 (14)	0.0484 (10)	−0.0223 (10)	−0.0131 (9)	0.0024 (10)
C36	0.0689 (11)	0.0618 (12)	0.0499 (11)	−0.0145 (9)	−0.0128 (9)	−0.0071 (9)
C37	0.0480 (10)	0.0512 (10)	0.0454 (9)	−0.0084 (8)	−0.0091 (8)	−0.0029 (8)
C38	0.0478 (10)	0.0633 (12)	0.0664 (11)	−0.0100 (8)	−0.0120 (8)	−0.0128 (9)
C39	0.0707 (13)	0.0675 (13)	0.0717 (12)	−0.0191 (10)	−0.0167 (10)	−0.0142 (10)
C40	0.0905 (15)	0.0592 (13)	0.0564 (11)	−0.0060 (11)	−0.0071 (11)	−0.0150 (9)
C41	0.0582 (12)	0.0847 (15)	0.0670 (13)	0.0043 (10)	0.0001 (10)	−0.0133 (11)
C42	0.0472 (11)	0.0827 (14)	0.0636 (12)	−0.0117 (9)	−0.0085 (9)	−0.0132 (10)
C22	0.0523 (10)	0.0564 (12)	0.0525 (10)	−0.0151 (8)	−0.0115 (8)	−0.0063 (9)
C23	0.0423 (9)	0.0534 (11)	0.0471 (10)	−0.0100 (7)	−0.0103 (7)	−0.0034 (8)
C28	0.0608 (10)	0.0501 (11)	0.0550 (11)	−0.0140 (8)	−0.0135 (8)	−0.0085 (8)
C27	0.0596 (11)	0.0573 (12)	0.0528 (11)	−0.0108 (8)	−0.0156 (8)	−0.0004 (9)
C26	0.0565 (10)	0.0632 (12)	0.0516 (10)	−0.0047 (8)	−0.0154 (8)	−0.0081 (9)
C25	0.0686 (12)	0.0593 (12)	0.0596 (12)	0.0005 (9)	−0.0171 (9)	−0.0163 (9)
C24	0.0625 (11)	0.0524 (11)	0.0598 (11)	−0.0024 (8)	−0.0192 (9)	−0.0048 (9)
C29	0.1421 (19)	0.0656 (14)	0.0724 (13)	−0.0160 (12)	−0.0396 (13)	0.0063 (11)
C30	0.1113 (16)	0.0927 (16)	0.0646 (12)	−0.0006 (12)	−0.0351 (12)	−0.0180 (11)

### Geometric parameters (Å, °)

**Table:**

N1—C1	1.2773 (19)	N3—C22	1.2797 (18)
N1—N2	1.3765 (17)	N3—N4	1.3701 (16)
N2—C16	1.405 (2)	N4—C31	1.4041 (19)
N2—C10	1.434 (2)	N4—C37	1.4362 (18)
C16—C21	1.381 (2)	C31—C36	1.386 (2)
C16—C17	1.388 (2)	C31—C32	1.389 (2)
C21—C20	1.376 (2)	C32—C33	1.376 (2)
C21—H21	0.93	C32—H32	0.93
C20—C19	1.369 (3)	C33—C34	1.374 (2)
C20—H20	0.93	C33—H33	0.93
C19—C18	1.364 (3)	C34—C35	1.371 (2)
C19—H19	0.93	C34—H34	0.93
C18—C17	1.379 (2)	C35—C36	1.377 (2)
C18—H18	0.93	C35—H35	0.93
C17—H17	0.93	C36—H36	0.93
C10—C15	1.360 (2)	C37—C38	1.373 (2)
C10—C11	1.374 (2)	C37—C42	1.374 (2)
C11—C12	1.374 (3)	C38—C39	1.373 (2)
C11—H11	0.93	C38—H38	0.93
C12—C13	1.350 (3)	C39—C40	1.366 (2)
C12—H12	0.93	C39—H39	0.93
C13—C14	1.347 (3)	C40—C41	1.368 (2)
C13—H13	0.93	C40—H40	0.93
C14—C15	1.379 (3)	C41—C42	1.374 (2)
C14—H14	0.93	C41—H41	0.93
C15—H15	0.93	C42—H42	0.93
C1—C2	1.455 (2)	C22—C23	1.455 (2)
C1—H1	0.93	C22—H22	0.93
C2—C3	1.387 (2)	C23—C24	1.390 (2)
C2—C7	1.390 (2)	C23—C28	1.390 (2)
C7—C6	1.377 (2)	C28—C27	1.390 (2)
C7—H7	0.93	C28—H28	0.93
C6—C5	1.381 (2)	C27—C26	1.396 (2)
C6—H6	0.93	C27—C29	1.509 (2)
C5—C4	1.397 (2)	C26—C25	1.384 (2)
C5—C9	1.506 (2)	C26—C30	1.509 (2)
C4—C3	1.387 (2)	C25—C24	1.375 (2)
C4—C8	1.504 (2)	C25—H25	0.93
C3—H3	0.93	C24—H24	0.93
C8—H8A	0.96	C29—H29A	0.96
C8—H8B	0.96	C29—H29B	0.96
C8—H8C	0.96	C29—H29C	0.96
C9—H9A	0.96	C30—H30A	0.96
C9—H9B	0.96	C30—H30B	0.96
C9—H9C	0.96	C30—H30C	0.96
			
C1—N1—N2	120.22 (14)	C22—N3—N4	120.47 (13)
N1—N2—C16	116.03 (14)	N3—N4—C31	116.75 (12)
N1—N2—C10	122.07 (14)	N3—N4—C37	122.52 (13)
C16—N2—C10	121.73 (13)	C31—N4—C37	120.46 (12)
C21—C16—C17	118.38 (17)	C36—C31—C32	118.44 (15)
C21—C16—N2	121.21 (15)	C36—C31—N4	120.01 (15)
C17—C16—N2	120.41 (16)	C32—C31—N4	121.54 (14)
C20—C21—C16	120.14 (18)	C33—C32—C31	119.90 (15)
C20—C21—H21	119.9	C33—C32—H32	120
C16—C21—H21	119.9	C31—C32—H32	120
C19—C20—C21	121.4 (2)	C34—C33—C32	121.46 (16)
C19—C20—H20	119.3	C34—C33—H33	119.3
C21—C20—H20	119.3	C32—C33—H33	119.3
C18—C19—C20	118.7 (2)	C35—C34—C33	118.75 (16)
C18—C19—H19	120.7	C35—C34—H34	120.6
C20—C19—H19	120.7	C33—C34—H34	120.6
C19—C18—C17	120.99 (18)	C34—C35—C36	120.70 (16)
C19—C18—H18	119.5	C34—C35—H35	119.7
C17—C18—H18	119.5	C36—C35—H35	119.7
C18—C17—C16	120.36 (18)	C35—C36—C31	120.73 (16)
C18—C17—H17	119.8	C35—C36—H36	119.6
C16—C17—H17	119.8	C31—C36—H36	119.6
C15—C10—C11	119.31 (17)	C38—C37—C42	119.71 (15)
C15—C10—N2	120.53 (17)	C38—C37—N4	119.82 (13)
C11—C10—N2	120.16 (17)	C42—C37—N4	120.44 (14)
C10—C11—C12	120.12 (18)	C39—C38—C37	120.19 (15)
C10—C11—H11	119.9	C39—C38—H38	119.9
C12—C11—H11	119.9	C37—C38—H38	119.9
C13—C12—C11	120.0 (2)	C40—C39—C38	120.10 (17)
C13—C12—H12	120	C40—C39—H39	120
C11—C12—H12	120	C38—C39—H39	120
C14—C13—C12	120.2 (2)	C39—C40—C41	119.84 (17)
C14—C13—H13	119.9	C39—C40—H40	120.1
C12—C13—H13	119.9	C41—C40—H40	120.1
C13—C14—C15	120.7 (2)	C40—C41—C42	120.45 (16)
C13—C14—H14	119.7	C40—C41—H41	119.8
C15—C14—H14	119.7	C42—C41—H41	119.8
C10—C15—C14	119.66 (19)	C37—C42—C41	119.70 (16)
C10—C15—H15	120.2	C37—C42—H42	120.1
C14—C15—H15	120.2	C41—C42—H42	120.1
N1—C1—C2	120.15 (16)	N3—C22—C23	120.75 (15)
N1—C1—H1	119.9	N3—C22—H22	119.6
C2—C1—H1	119.9	C23—C22—H22	119.6
C3—C2—C7	117.54 (14)	C24—C23—C28	117.36 (14)
C3—C2—C1	120.02 (15)	C24—C23—C22	122.72 (15)
C7—C2—C1	122.44 (15)	C28—C23—C22	119.89 (15)
C6—C7—C2	120.26 (15)	C27—C28—C23	122.90 (15)
C6—C7—H7	119.9	C27—C28—H28	118.6
C2—C7—H7	119.9	C23—C28—H28	118.6
C7—C6—C5	121.94 (15)	C28—C27—C26	118.71 (15)
C7—C6—H6	119	C28—C27—C29	119.93 (15)
C5—C6—H6	119	C26—C27—C29	121.33 (15)
C6—C5—C4	118.81 (14)	C25—C26—C27	118.42 (14)
C6—C5—C9	120.28 (14)	C25—C26—C30	119.86 (16)
C4—C5—C9	120.90 (14)	C27—C26—C30	121.70 (16)
C3—C4—C5	118.58 (15)	C24—C25—C26	122.32 (16)
C3—C4—C8	120.75 (15)	C24—C25—H25	118.8
C5—C4—C8	120.67 (14)	C26—C25—H25	118.8
C4—C3—C2	122.85 (15)	C25—C24—C23	120.28 (15)
C4—C3—H3	118.6	C25—C24—H24	119.9
C2—C3—H3	118.6	C23—C24—H24	119.9
C4—C8—H8A	109.5	C27—C29—H29A	109.5
C4—C8—H8B	109.5	C27—C29—H29B	109.5
H8A—C8—H8B	109.5	H29A—C29—H29B	109.5
C4—C8—H8C	109.5	C27—C29—H29C	109.5
H8A—C8—H8C	109.5	H29A—C29—H29C	109.5
H8B—C8—H8C	109.5	H29B—C29—H29C	109.5
C5—C9—H9A	109.5	C26—C30—H30A	109.5
C5—C9—H9B	109.5	C26—C30—H30B	109.5
H9A—C9—H9B	109.5	H30A—C30—H30B	109.5
C5—C9—H9C	109.5	C26—C30—H30C	109.5
H9A—C9—H9C	109.5	H30A—C30—H30C	109.5
H9B—C9—H9C	109.5	H30B—C30—H30C	109.5
			
C1—N1—N2—C16	179.59 (15)	C22—N3—N4—C31	−175.97 (13)
C1—N1—N2—C10	−5.1 (2)	C22—N3—N4—C37	−1.9 (2)
N1—N2—C16—C21	−8.0 (2)	N3—N4—C31—C36	168.14 (13)
C10—N2—C16—C21	176.64 (17)	C37—N4—C31—C36	−6.0 (2)
N1—N2—C16—C17	172.04 (15)	N3—N4—C31—C32	−12.63 (19)
C10—N2—C16—C17	−3.3 (3)	C37—N4—C31—C32	173.21 (13)
C17—C16—C21—C20	0.2 (3)	C36—C31—C32—C33	1.5 (2)
N2—C16—C21—C20	−179.79 (16)	N4—C31—C32—C33	−177.73 (13)
C16—C21—C20—C19	−0.6 (3)	C31—C32—C33—C34	−0.6 (2)
C21—C20—C19—C18	0.3 (3)	C32—C33—C34—C35	−0.5 (2)
C20—C19—C18—C17	0.4 (3)	C33—C34—C35—C36	0.6 (2)
C19—C18—C17—C16	−0.8 (3)	C34—C35—C36—C31	0.3 (2)
C21—C16—C17—C18	0.5 (3)	C32—C31—C36—C35	−1.4 (2)
N2—C16—C17—C18	−179.52 (15)	N4—C31—C36—C35	177.86 (14)
N1—N2—C10—C15	−86.5 (2)	N3—N4—C37—C38	103.54 (17)
C16—N2—C10—C15	88.6 (2)	C31—N4—C37—C38	−82.65 (18)
N1—N2—C10—C11	93.2 (2)	N3—N4—C37—C42	−78.19 (18)
C16—N2—C10—C11	−91.7 (2)	C31—N4—C37—C42	95.62 (18)
C15—C10—C11—C12	−1.7 (3)	C42—C37—C38—C39	−1.1 (2)
N2—C10—C11—C12	178.59 (16)	N4—C37—C38—C39	177.16 (15)
C10—C11—C12—C13	1.0 (3)	C37—C38—C39—C40	0.9 (3)
C11—C12—C13—C14	0.1 (3)	C38—C39—C40—C41	−0.1 (3)
C12—C13—C14—C15	−0.5 (3)	C39—C40—C41—C42	−0.6 (3)
C11—C10—C15—C14	1.3 (3)	C38—C37—C42—C41	0.4 (2)
N2—C10—C15—C14	−178.98 (17)	N4—C37—C42—C41	−177.84 (15)
C13—C14—C15—C10	−0.2 (3)	C40—C41—C42—C37	0.5 (3)
N2—N1—C1—C2	179.62 (14)	N4—N3—C22—C23	177.09 (12)
N1—C1—C2—C3	176.21 (15)	N3—C22—C23—C24	−1.1 (2)
N1—C1—C2—C7	−2.8 (2)	N3—C22—C23—C28	−178.83 (13)
C3—C2—C7—C6	0.9 (2)	C24—C23—C28—C27	−0.9 (2)
C1—C2—C7—C6	179.98 (15)	C22—C23—C28—C27	176.88 (14)
C2—C7—C6—C5	−0.7 (2)	C23—C28—C27—C26	0.9 (2)
C7—C6—C5—C4	−0.4 (2)	C23—C28—C27—C29	−177.19 (15)
C7—C6—C5—C9	178.62 (14)	C28—C27—C26—C25	0.2 (2)
C6—C5—C4—C3	1.2 (2)	C29—C27—C26—C25	178.17 (16)
C9—C5—C4—C3	−177.80 (14)	C28—C27—C26—C30	−178.03 (16)
C6—C5—C4—C8	−178.79 (15)	C29—C27—C26—C30	0.0 (2)
C9—C5—C4—C8	2.2 (2)	C27—C26—C25—C24	−1.1 (2)
C5—C4—C3—C2	−1.0 (2)	C30—C26—C25—C24	177.13 (16)
C8—C4—C3—C2	179.01 (15)	C26—C25—C24—C23	1.0 (2)
C7—C2—C3—C4	−0.1 (2)	C28—C23—C24—C25	0.0 (2)
C1—C2—C3—C4	−179.15 (14)	C22—C23—C24—C25	−177.75 (14)

### Hydrogen-bond geometry (Å, °)

**Table:**

Cg1, Cg2, Cg3 and Cg4 are the centroids of the C31–C36, C37–C42, C2–C7 and C23–C28 rings, respectively.

**Table:**

*D*—H···*A*	*D*—H	H···*A*	*D*···*A*	*D*—H···*A*
C3—H3···Cg1^i^	0.93	2.81	3.7152 (18)	166
C6—H6···Cg1^ii^	0.93	2.66	3.5506 (19)	160
C9—H9C···Cg2^iii^	0.96	2.97	3.7170 (19)	136
C29—H29B···Cg3^iv^	0.96	3.00	3.931 (2)	165
C41—H41···Cg4^i^	0.93	2.83	3.590 (2)	139

Symmetry codes: (i) −*x*+1, −*y*, −*z*; (ii) −*x*, −*y*+1, −*z*; (iii) *x*, *y*+1, *z*; (iv) *x*, *y*−1, *z*.
